# C5aR1 shapes a non-inflammatory tumor microenvironment and mediates immune evasion in gastric cancer

**DOI:** 10.17305/bjbms.2022.8317

**Published:** 2023-05-01

**Authors:** Honghong Shen, Xianhua Gu, Xinwei Li, Zheng Xiang, Rong Zhang, Fan Shi, Mingyue Tang, Huiyuan Li, Guangzheng Zhu, Fang Su, Zishu Wang

**Affiliations:** 1 Department of Medical Oncology, First Affiliated Hospital of Bengbu Medical College, Bengbu, China; 2 Department of Gynecology Oncology, First Affiliated Hospital of Bengbu Medical College, Bengbu, China; 3 Department of Surgical Oncology, First Affiliated Hospital of Bengbu Medical College, Bengbu, China; 4 Department of Pathology, First Affiliated Hospital of Bengbu Medical College, Bengbu, China

**Keywords:** *C5aR1*, gastric cancer (GC), immune escape, immunotherapy, tumor microenvironment

## Abstract

C5a receptor 1 (C5aR1) is associated with various inflammatory processes, the pathogenesis of immune diseases, and tumor growth. However, its role in the tumor microenvironment of gastric cancer (GC) remains unclear. In this study, the expression of *C5aR1* in GC and normal gastric mucosa tissues was compared using data retrieved from the Gene Expression Omnibus (GEO) and The Cancer Genome Atlas (TCGA) databases, and the results were validated by in vitro qRT-PCR and immunohistochemical analyses. The relationship between *C5aR1* expression and the overall survival of patients with GC was analyzed using the Kaplan–Meier method. Subsequently, enrichment analysis was performed, and the signaling pathways were screened. *C5aR1* expression was also correlated with genes related to the immune checkpoint and immune cell infiltration. The results revealed that *C5aR1* expression was enhanced in GC tissues compared to normal gastric tissues, and that patients with high expression of *C5aR1* had a worse 10-year overall survival compared to those showing low expression of *C5aR1*. Functional analysis revealed that *C5aR1* is a gene related to the immune system and may play a crucial role in inflammatory and tumor immune responses. Additionally, *C5aR1* showed a positive correlation with most immune checkpoint-related genes and a negative correlation with natural killer cells, dendritic cells, and CD8+ T cells. Immune evasion risk was observed to be significantly greater in patients with higher expression of *C5aR1* than in those with lower expression. The results of this study reveal that *C5aR1* shapes a non-inflammatory tumor microenvironment in GC and mediates immune evasion.

## Introduction

C5a receptor 1 (C5aR1), a membrane-bound G protein-coupled receptor (GPCR), is the most commonly studied C5a receptor [1]. The complement system is an important constituent of the innate immune response, and it regulates humoral immunity and pathogen immune surveillance and maintains tissue homeostasis [2, 3]. The C5a/C5aR1 activation pathway has been implicated in various inflammatory processes, the pathogenesis of immune diseases, and tumor growth [4]. Many studies have suggested the use of complement inhibitors as a new therapeutic method for various cancers and described their clinical applications [5, 6].

Gastric cancer (GC) is the fifth most prevalent cancer and the fourth leading cancer-related cause of death globally. In 2020, approximately 769,000 deaths due to GC were reported [7]. In addition to chemotherapy, radiation, gastrectomy, and targeted therapy, immunotherapy is also used to treat GC [8]. However, despite the recent breakthroughs in therapeutic techniques, the mortality rate of GC remains high [9].

Blocking programmed cell death protein 1 (PD-1)/PD-1 ligand 1 (PD-L1) and cytotoxic T lymphocyte-associated protein 4 (CTLA-4), immune checkpoints using antibodies is a highly effective immunotherapeutic strategy for cancer. Patients with melanoma, glioma, and Hodgkin’s lymphoma have benefited considerably from immune checkpoint inhibition in the last few decades [10]. Immune checkpoint inhibitor therapy provides a modest survival benefit in GC, with anti-PD-1 therapy improving overall survival to 12–18 months [11]. However, the treatment outcomes remain unsatisfactory. Current evidence suggests that the tumor microenvironment (TME) for the survival and growth of tumor cells is considerably involved in tumor progression. In addition to malignant cells, the TME comprises adipocytes, fibroblasts, tumor vascular system, lymphocytes, dendritic cells, and cancer-associated fibroblasts. The unique immune capabilities of these cells can affect the survival of tumor cells and influence the neighboring cells [12]. Therefore, addressing the TME not only complements but also improves the outcome of cancer treatment [13]. In our previous study, we found that complement activation in TME enhances tumor growth and metastasis [14]. Cancer cells can enhance proliferation and angiogenesis by interacting directly or indirectly with TME components, thus preventing apoptosis, avoiding hypoxia, and developing immunological tolerance. Recent studies have revealed the importance of TME in tumor growth, immune escape, and immunotherapeutic responses. Therefore, the characterization of cell infiltration in TME is critical for predicting the response to immune checkpoint blockade therapy and improving the success rate of the therapy [15, 16].

In this study, we investigated the *C5aR1* expression in a GC cohort and its relationship with prognosis. We further evaluated the association of the expression of *C5aR1* with immune checkpoints and immune cell infiltration. In addition, *C5aR1’s* biological function in GC was determined using gene set enrichment analysis (GSEA). The results demonstrate that *C5aR1* shapes a non-inflammatory TME in GC and mediates immune evasion.

**Table 1:** The information on the utilized GEO datasets in our study

**Dataset**	**Topics**	**Number of samples**
GSE13911	The expression profiles of both primary gastric tumors (MSI and MSS) and adjacent normal samples	38 gastric tumors 31 normal gastric tissues
GSE29272	Data of affymetrix gene expression array for cardia and non-cardia gastric cancer samples	134 gastric tumors 134 normal gastric tissues
GSE54129	Global gene expression assessment of gastric cancer by oligonucleotide microarrays	21 gastric tumors 111 normal gastric tissues

MSI: Microsatellite instable; MSS: Microsatellite stable; GEO: Gene Expression Omnibus.

## Materials and methods

### Gastric cancer datasets and pre-processing

Common gene expression data and clinical data on mortality and prognosis of GC patients were retrieved from the Gene Expression Omnibus (GEO) database (http://www.ncbi.nlm.nih.gov/geo/) and The Cancer Genome Atlas (TCGA) database [17, 18]. The GEO datasets GSE13911, GSE29272, and GSE54129 were used for analysis (Table 1). By integrating the multi-array averaging methods of simpleaffy and Affy, we adjusted the background and performed quantile normalization. TCGA biolink, an R program, was utilized to perform a comprehensive analysis of RNA-sequencing (fragments per kilobase of transcript per million reads mapped [FPKM] values) and data related to cytogenetic mutations obtained from TCGA. The values of FPKM were translated to the transcripts per kilobase million. To correct the non-biological technical bias batch effect, the sva package’s “ComBat” algorithm was utilized. Analyses of all data were done in R (v4.1.2) with the help of the R-Bioconductor software.

### Collection of clinical samples

Between January and December 2017, tissue samples were obtained from patients diagnosed with GC who received surgical treatment at the Department of Gastrointestinal Surgery at the First Affiliated Hospital of Bengbu Medical College. For quantitative real-time polymerase chain reaction (qRT-PCR), 10 GC and adjoining normal tissue samples were used, while for immunohistochemistry (IHC) analysis, 62 GC and 10 adjoining normal tissue samples were used. Patients did not receive radiotherapy, chemotherapy, or biological treatment pre- or postoperatively nor had they been diagnosed with GC before. The postoperative sample tissues were frozen at −80 ^∘^C for the extraction of proteins.

### Experimental materials

Rabbit anti-human C5aR1 antibodies were purchased from Abcam (EPR23278-117, ab252435) (40 µL Cambridge, UK). Primary antibodies against actin and alpha rabbit monoclonal antibodies targeting CD3+/CD4+/CD8+ T cells were supplied from Cell Signaling Technology Inc. (Danvers, MA, USA). Jackson ImmunoResearch Inc. (West Grove, PA, USA) supplied a rabbit anti-mouse antibody coupled with horseradish peroxidase. Albumin from bovine serum was acquired from Sigma-Aldrich (St. Louis, MO, USA). Sangon Biotech Co., Ltd. (Shanghai, China) supplied the skim milk and Tween-20 used in this experiment. Thermo Fisher Scientific (USA) provided the TRIzol reagent. Two Japanese companies, TaKaRa (Tokyo) and TOYOBO (Osaka), provided the following reagents used, respectively: the PrimeScriptTM First Strand cDNA Synthesis Kit and the SYBR Green Real-Time PCR Master Mix.

### Immunohistochemical analysis

Paraformaldehyde (4%) was used to fix the tissue samples, which were then encased in paraffin, cut into slices (4 µm), and adhered to slides. Various xylene density gradients were used to deparaffinize the samples before rehydration. After incubating the samples at 82 ^∘^C for 24 min, antigens were extracted in a citric acid buffer (0.1 M; pH 7.8). The peroxidase activity on the slides was then inhibited by exposing them to a peroxidase-blocking buffer at room temperature for 15 min. Following that, the slides were subjected to incubation for an entire night with the presence of an anti-C5aR1, anti-CD3, anti-CD4, and anti-CD8 antibody mixture. The slides were subjected to gentle washing with phosphate-buffered saline the next day, after which they were treated with a biotin-conjugated secondary antibody for 10 min at room temperature, with subsequent treatment for 5 min with streptavidin peroxidase. Afterward, the samples were hematoxylin-stained, rinsed to eliminate any residual debris, and then dried in the air in preparation for the IHC examination.

### Reverse transcription polymerase chain reaction quantification

With the use of the TRIzol reagent, total RNA was isolated. The RNA that was obtained was then subjected to reverse transcription with the aid of the RevertAid First Strand cDNA Synthesis Kit, and the cDNA that was synthesized was then extracted using the SYBR Green Realtime PCR Master Mix. We used the following primers for qRT-PCR: human *C5aR1* forward, 5’-CGGGAGGAGTACTTTCCACC-3’; *C5aR1* reverse, 5’-GTGCTCACGGCGGCTC-3’; GAPDH forward, 5’-AAGGTGTTCTECTCGGTGAC-3’; GAPDH reverse, 5’-GAGGGTAGAGGACTGAATAGTACCTG-3’. The internal control for the study was GAPDH. There were three rounds of testing for every single sample from every group. To examine the results of the qRT-PCR experiment, a paired Student’s *t*-test was carried out.

### Nomogram development and validation for GC patients

Prognostic variables were derived from univariate and multivariate analyses, and a nomogram model was developed to anticipate one-, three-, and five-year overall survival (OS) for GC patients. Nomogram analysis was conducted with the “RMS” tool in R. Calibration curves were employed to visually compare the nomogram’s predicted probability (using the Kaplan–Meier analysis) with the actual values using the Kaplan–Meier analysis. Nomogram prediction models that have been calibrated correctly will have their scatter points cluster along a 45∘ diagonal line. Harrell concordance index (C-index) was also utilized to examine the nomogram model’s overall prediction ability. Prediction accuracy was measured by the C-index, ranging between 0.5 and 1, with a higher C-index indicating superior predictive efficacy. Two-tailed tests were performed on all data in this study, and a value of 0.05 indicated a significance level for all comparisons.

### Functional analyses of *C5aR1* in GC

The clusterProfiler software was utilized to perform the Kyoto Encyclopedia of Genes and Genomes (KEGG) as well as Gene Ontology (GO) functional enrichment analyses to better understand the roles played by *C5aR1* in GC. The three facets of enrichment of GO, i.e., cellular components, molecular processes, and biological functions, were demonstrated. The clusterProfiler program was also used to conduct enrichment analyses of differentially expressed genes (DEGs), as well as the important signaling pathways associated with the DEGs were evaluated using a bubble graph. The genes used as candidates for GSEA were classified into high- and low-risk groups as per their mean risk score. Moreover, the datasets of functional predefined genes were derived from the MsigDB Database (molecular signatures database/repository). To determine whether a candidate gene for a pathway was significantly enriched (*P* < 0.05), a false discovery rate of 0.25 was applied as the screening criteria. The signaling pathways with substantial enrichment were chosen using a combination of the adjusted *P* value and normalized enrichment score.

### Evaluation of the immunological features of the GC tumor microenvironment

The “preprocessCore” program and CIBERSORT technique were adopted to assess the infiltration status of 22 distinct immune cells to assess the link between the *C5aR1* gene expression and immune cell infiltration [19]. Furthermore, TCGA database was employed to probe the link between the *C5aR1* expression and 22 immune checkpoint molecules. A *P* value of < 0.05 denoted the significance threshold.

### Ethical statement

The First Affiliated Hospital of Bengbu Medical College’s Ethics Committee granted its approval to this research involving human subjects ([2022]298). Patients/participants gave their consent to be involved in this research through the signing of a consent form.

### Statistical analysis

In this study, GSEA (v 4.2.3), Perl (v 5.32.1.1), and R software (v 4.1.2) were employed to analyze the data. Throughout the investigation, all statistical methodologies and appropriate R software packages were described. The cutoff criterion was established at *P* < 0.05.

## Results

### Expression of *C5aR1* was elevated in human GC tissues

**Figure 1.**
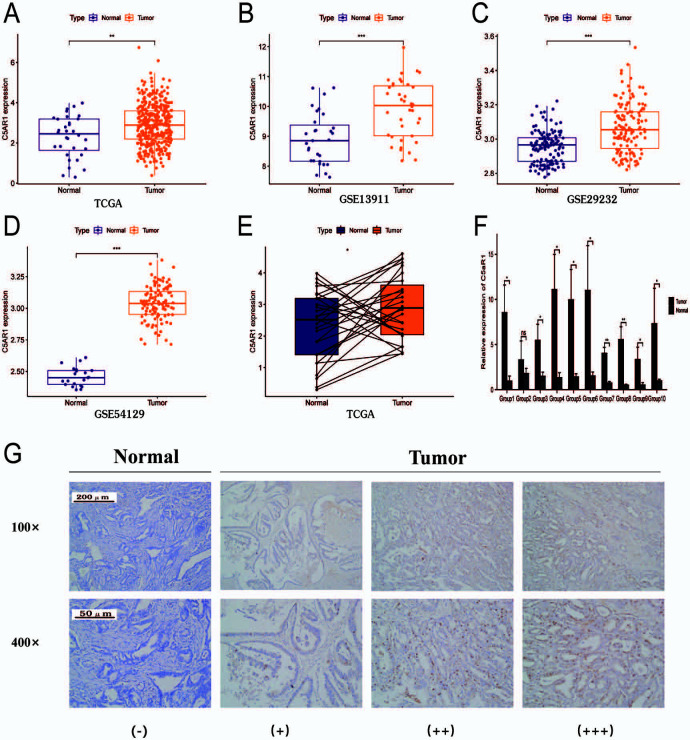
**The expression of *C5aR1* in gastric cancer tissues and adjacent normal tissues.** (A) *C5aR1* expression was upregulated in GC tissues in the TCGA cohort. (B)–(D) *C5aR1* expression was enhanced in GC tissues in the (B) GSE13911, (C) GSE29272, and (D) GSE54129 datasets; (E) *C5aR1* was overexpressed in GC tissues in contrast to the paraneoplastic tissues in the TCGA cohort; (F) qRT-PCR depicted that expression of *C5aR1* was significantly enhanced in GC tissues compared to normal tissues; (G) Immunohistochemical assessment showed that expression of C5aR1 was significantly enhanced in GC tissues compared to adjacent normal tissues (*P < 0.05; **P < 0.01; ***P < 0.001). C5aR1: C5a receptor 1; GC: Gastric cancer; TCGA: The Cancer Genome Atlas**;** qRT-PCR: Quantitative real-time polymerase chain reaction.

*C5aR1* mRNA expression was enhanced in GC tissues as compared to adjacent normal gastric tissues in TCGA cohort (*P* < 0.05; Figure 1A). This result was verified using data from three GEO datasets, namely, GSE13911, GSE29272, and GSE54129 (*P* < 0.05; Figure 1B–1D). In addition, a comparison of *C5aR1* mRNA expression in 32 pairs of tumors and adjoining normal tissues in the database of TCGA revealed that its expression level was elevated in tumor tissues compared to adjoining normal tissues (*P* < 0.05; Figure 1E). Similarly, the qRT-PCR analysis showed a higher *C5aR1* mRNA expression in the tissues of GC in comparison to the normal tissues (*P* < 0.05; Figure 1F). IHC staining also showed that levels of C5aR1 were elevated in tissues of GC as compared to paraneoplastic tissues (Figure 1G).

### Prognostic analysis of *C5aR1* in GC

**Figure 2.**
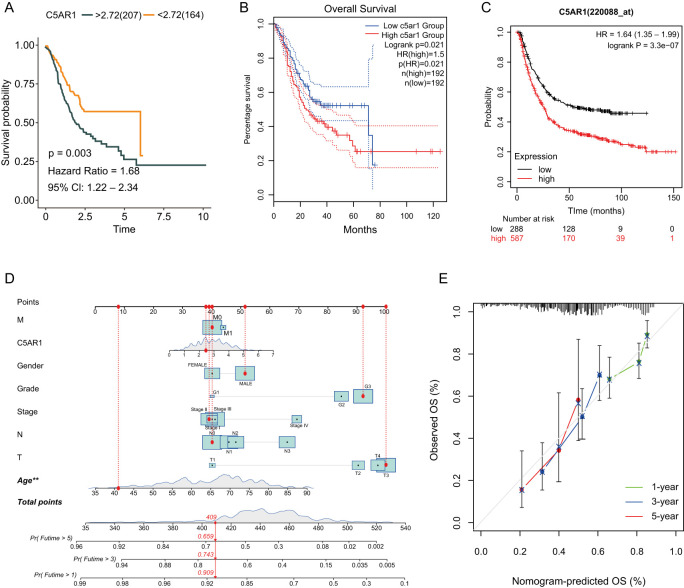
**Prognostic analysis of *C5aR1* in gastric cancer.** (A) Survival curve demonstrating the impact of *C5aR1* expression on OS in TCGA cohort; (B) Survival curve showing the impact of *C5aR1* expression on OS based on data extracted from the GEPIA database; (C) Survival curve showing the impact of *C5aR1* expression on OS in the Kaplan–Meier Plotter database; (D) Nomogram for predicting one-, three- and five-year OS rates of patients with gastric cancer; (E) Nomogram model calibration curves for predicting one-, three-, and five-year OS rates. OS: Overall survival; C5aR1: C5a receptor 1; TCGA: The Cancer Genome Atlas; HR: Hazard ratio; CI: Confidence interval.

The Kaplan–Meier approach was utilized to analyze the patients’ survival with various levels of *C5aR1* expression using data from TCGA and two online databases, thus assessing the prognostic potential of *C5aR1* in GC. The results revealed that 10-year OS was better in patients with low *C5aR1* expression levels compared to those with high expression levels (*P* < 0.05; Figure 2A–2C). A nomogram was developed to predict GC patients’ prognosis status (Figure 2D). Variables for the nomogram were selected on the basis of findings of multivariate and univariate regression. Each variable had a point value determined using a 100-point scale. The overall prone score, which ranged from 0 to 240 points, was computed by adding all variable points. To estimate the one-, three-, and five-year survival probability of GC individuals, a vertical line was drawn from the total point scale on the survival probability lines. C-index and calibration curves were then used to further assess the accuracy and reliability of the nomogram in predicting survival for computational validation. The bias-corrected one-, three-, and five-year lines on the calibration plot were found to be relatively near to the standard 45∘ diagonal line, indicating that the theoretical and observed values were similar (Figure 2E). As shown herein, the nomogram model is effective in making predictions for one-, three-, and five-year OS of patients with GC.

### Functional analysis of *C5aR1* in GC

**Figure 3.**
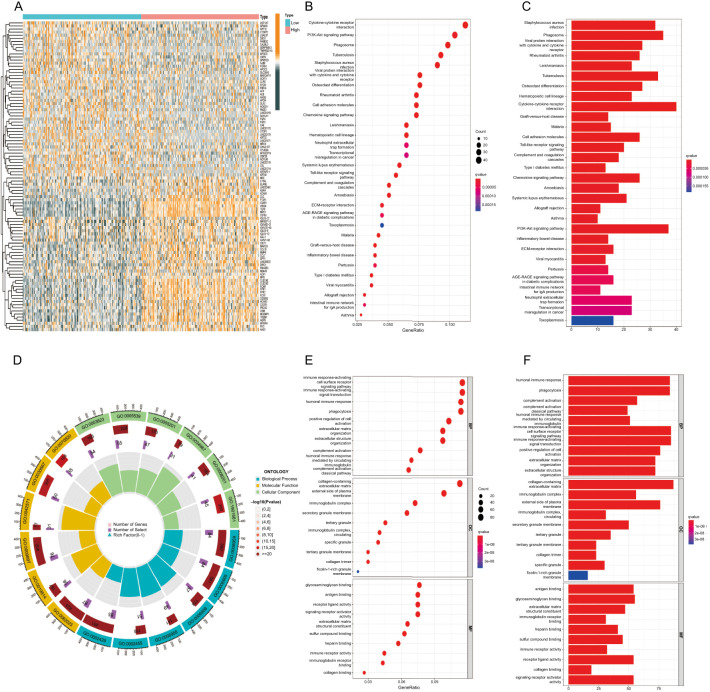
**Functional analysis of *C5aR1* in gastric cancer.** (A) Heatmap of differentially expressed DEGs between the high and low *C5aR1* expression groups. (B) and (C) *C5aR1*-related KEGG enrichment analysis (D)–(F) *C5aR1*-related GO function annotation. DEG: Differentially expressed genes; C5aR1: C5a receptor 1; GO: Gene Ontology; KEGG: Kyoto Encyclopedia of Genes and Genomes.

We divided GC samples into two categories as per *C5aR1* expression (high and low). Subsequently, we examined and obtained the DEGs in the two categories (fdrFilter ═ 0.05). We obtained 1046 differential genes and plotted a differential gene heat map (Figure 3A). KEGG and GO analyses were then used to resolve the function of these genes. The KEGG assessment highlighted that the enrichment of DEGs occurred in pathways associated with cytokine–cytokine receptor interaction and PI3K–Akt signaling (Figure 3B and 3C). It was shown by the GO analysis that enrichment of DEGs occurred in the biological processes such as the immune response-activating cell surface receptor signaling pathway, cellular components such as the external side of the plasma membrane, collagen-containing extracellular matrix, and molecular functions such as glycosaminoglycan and antigen binding (Figure 3D–3F).

**Figure 4.**
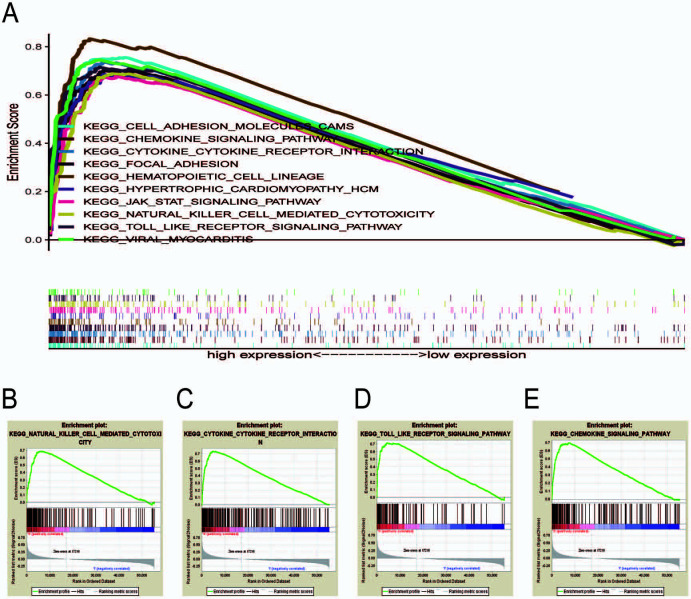
**Gene set enrichment analysis (GSEA).** (A) GSEA of the top 10 upregulated pathways associated with *C5aR1* in gastric cancer; (B) Natural killer cell-mediated cytotoxicity; (C) Cytokine receptor interaction; (D) Chemokine signaling pathway; (E) Toll-like receptor signaling pathway. C5aR1: C5a receptor 1.

GSEA was employed to screen for the top 10 signaling pathways associated with *C5aR1* in GC (Figure 4A). The results depicted that pathways linked to inflammatory and immune responses were subjected to enrichment in the high *C5aR1* expression group, including natural killer (NK) cell-mediated cytotoxicity, cytokine receptor interaction, chemokine signaling pathway, and toll-like receptor signaling pathway (Figure 4B–4E). Altogether, these results suggest that the *C5aR1* gene is related to the immune system that plays a key role in the inflammatory and tumor immune responses, promoting the carcinogenesis and progression of GC.

### Correlational analysis of *C5aR1* with 22 common immune checkpoint genes in GC

**Figure 5.**
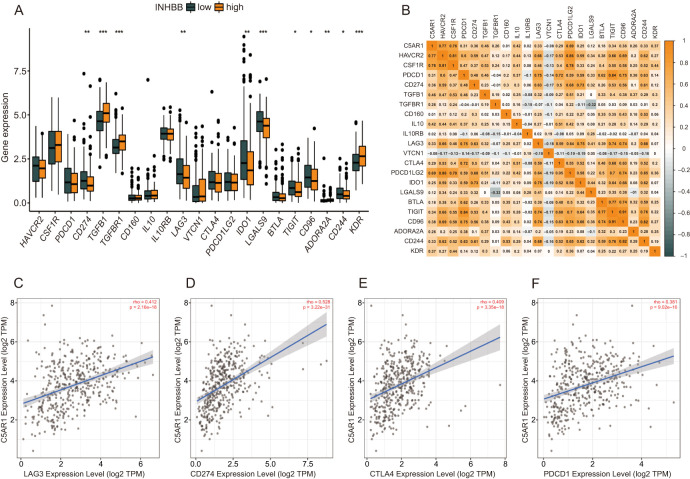
**Correlation between *C5aR1* expression and 22 common immune checkpoint-related genes in gastric cancer.** (A) Differential expression of 22 immune checkpoint-related genes in the high and low *C5aR1* expression groups; (B) Correlation between *C5aR1* and 22 immune checkpoint-related genes in gastric cancer; (C)–(F) Correlation between the expression of *C5aR1* and *LAG3*, *CD274*, *CTLA4,* and *PDCD1* was analyzed using TIMER. C5aR1: C5a receptor 1; TIMER: Tumor Immune Estimation Resource.

The correlation was analyzed between *C5aR1* and 22 immune checkpoint-related genes using the TCGA database and Tumor Immune Estimation Resource (TIMER) [20] (Figure 5). The immune checkpoint gene expression levels were remarkably higher in the high *C5aR1* expression score group compared to the low *C5aR*1 expression score group (Figure 5A). *LAG3*, *CD274*, *CTLA4,* and *PDCD1* correlated positively with *C5aR1* expression (Figure 5B–5F).

### *C5aR1* shapes a non-inflammatory tumor microenvironment and mediates immune evasion in GC

**Figure 6.**
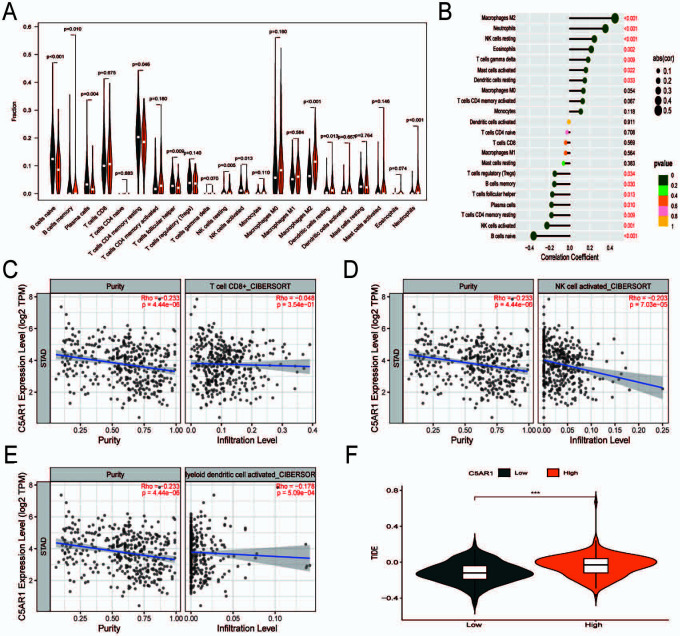
***C5aR1* shapes a non-inflammatory tumor microenvironment and mediates immune evasion in gastric cancer**. (A) Violin plot based on *C5aR1* expression analyses differences with 22 immune cell types in gastric cancer; (B) Correlation between *C5aR1* expression and 22 immune cell types in gastric cancer; (C)–(E) *C5aR1* expression showed a negative correlation with NK cells, CD8+ T cells, and dendritic cells; (F) Assessment of immune evasion efficacy in the high and low C5aR1 expression groups (*P < 0.05; **P < 0.01; ***P < 0.001). C5aR1: C5a receptor 1; NK: Natural killer.

The CIBERSORT tool was used to determine the link between the expression of *C5aR1* and immune cell infiltration. GC patients were classified into high and low *C5aR1* expression groups as per the median *C5aR1* expression. The abundance of 22 immune cell types was compared in the two groups (Figure 6A). *C5aR1* expression was inversely linked to naive B cells, activated NK cells, and most T cells (naive CD8+ and CD4+ T cells) and positively linked to M2 macrophages (Figure 6B). Analysis performed using TIMER [20] revealed that *C5aR1* expression was negatively correlated with CD8+ T cells, NK cells, and dendritic cells (Figure 6C–6E). In the previous studies, TME has been categorized into two subtypes: an inflammatory TME characterized by T cell infiltration and a non-inflammatory TME characterized by T cell suppression. Tumors with T cell inflammation contain abundant CD8α/CD103-lineage dendritic cells and CD8+ T cells. Tumors without T cell inflammation are devoid of these cells but possess fibroblasts, blood vessels, and macrophages, which enhance tumor growth [21, 22]. We speculate that *C5aR1* shapes a non-inflammatory TME (immune-exclusion phenotype) in GC. The Tumor Immune Dysfunction and Exclusion (TIDE) [23] scores were significantly greater in the group with high *C5aR1* expression as compared to the group with low *C5aR1* expression, which indicates that higher expression of *C5aR1* mediates the immune-evasion status of the tumor immune microenvironment (TIME) in GC (Figure 6F).

### Analyzing immune cell infiltration in groups with high and low C5aR1 expression

**Figure 7.**
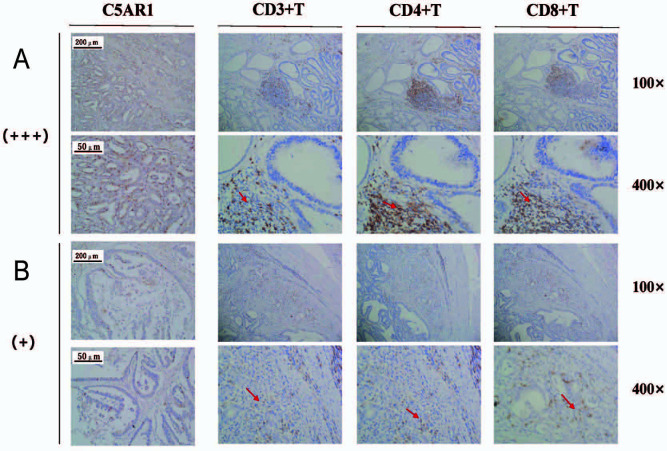
**High and low C5aR1 expression groups’ immune cell infiltration status.** (A) Infiltration of CD3+, CD4+, and CD8+ T cells in the high C5aR1 expression group (+++); (B) Infiltration of CD3+, CD4+, and CD8+ T cells in the low C5aR1 expression group (+). The red arrows indicate the location of immune cells. C5aR1: C5a receptor 1.

Given that *C5aR1* is negatively correlated with CD8+ and CD4+ T cells, we verified whether *C5aR1* is involved in immune evasion in the TIME of GC. Tissue samples with low (+) and high (+++) C5aR1 levels were analyzed via IHC staining (Figure 1G). The aim of IHC staining was to assess the CD4+ and CD8+ T cell infiltration in GC and surrounding tissues to evaluate the correlation between high C5aR1 expression and the features of immune evasion in TIME. The results revealed that CD4+ and CD8+ T cells in GC tissues with high (+++) C5aR1 levels were particularly clustered in the peripheral tumor stroma, with a small proportion of immune cells infiltrating the stroma of the parenchyma of tumor mass (Figure 7A). However, in GC tissues with low (+) C5aR1 levels, fewer CD8+ and CD4+ T cells were clustered in the peripheral tumor stroma, and a higher number of immune cells infiltrated the stroma of the tumor parenchyma (Figure 7B). This finding verified that a higher C5aR1 expression level leads to immune evasion in the TIME of GC.

### Expression of *C5aR1* in GC and evaluation of immunotherapy

**Figure 8.**
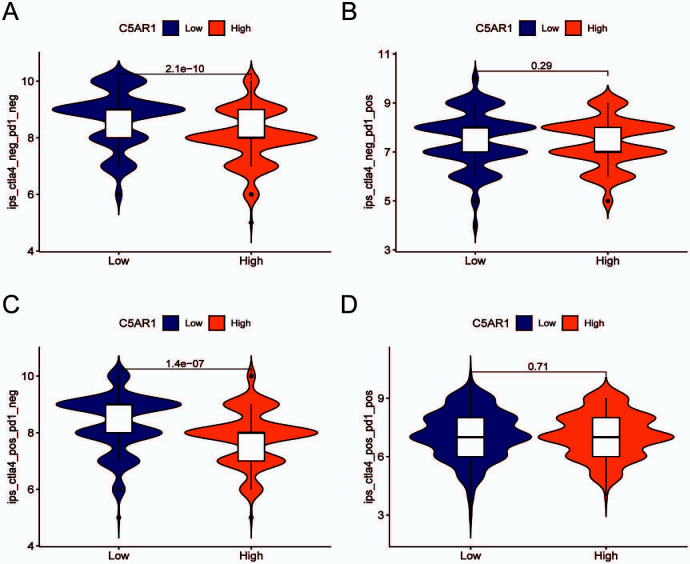
**Expression of *C5aR1* in gastric cancer and evaluation of immunotherapy.** (A)–(D) The relationship between IPS and the *C5aR1* expression in GC patients based on TCIA; CTLA-4- PD-1- (A), CTLA-4- PD-1+ (B), CTLA-4+ PD-1- (C), CTLA-4+PD-1+ (D). TCIA: The Cancer Immunome Database; PD-1: Programmed cell death protein 1; PD-L1: Programmed cell death protein ligand 1; CTLA-4: Cytotoxic T lymphocyte-associated protein 4; IPS: Immune phenomenon scores; C5aR1: C5a receptor 1.

After exploring immune phenomenon scores through The Cancer Immunome Database (TCIA) [24], we found that anti-CTLA-4 immunotherapy was better in the low *C5aR1* expression score group (Figure 8A–8D).

## Discussion

As an effector arm of innate and adaptive humoral immunity, the complement system regulates tissue homeostasis, enhances immune surveillance, and plays a vital role in the human immune system [2]. As an important part of the complement system, the C5a/C5aR1 activation pathway is implicated in various inflammatory processes, the pathogenesis of immune diseases, and tumor growth [4]. For example, activation of the complement components C5 and C5a in cancer tissues and expression of C5aR1 in breast cancer cells is linked to a poor prognosis. Moreover, various studies have shown that inhibition of the C5a/C5aR1 pathway can help in the management of breast cancer [25]. In addition, C5a is a potential prognostic biomarker for colon cancer, and targeting the C5aR1 pathway may help to treat colon cancer [26]. The complement C5a/C5aR1 pathway enhances the pathogenesis of GC by downregulating p21 expression [27]. However, the role of C5aR1 in the TME of GC has not been reported.

GC, one of the most prevalent gastrointestinal tract tumors, is the fourth contributor to cancer-related deaths globally and ranks fifth in terms of global cancer incidence [28, 29]. However, the 5-year survival rate for subjects with early diagnosis and treatment is approximately 90%–97% and less than 30% for individuals with GC who experience advanced or metastatic disease [28, 29]. Gene therapy, molecular targeted therapy, and cancer immunotherapy are currently being used in the treatment and diagnosis of GC, and the patients’ prognosis with advanced GC remains extremely poor [30, 31]. TME has become a potential therapeutic target for various malignancies, including GC, because of its key contribution to cancer proliferation and resistance to medicines [32]. Patients with advanced HER2-positive GC have had trastuzumab plus chemotherapy as their first-line treatment option since the ToGA trial in 2010 [33]. OS was improved in HER2-positive patients treated in the first line with trastuzumab plus cisplatin and fluoropyrimidine, despite the fact that all patients with advanced disease experienced disease progression following first- and second-line treatment [34]. Multiple types of solid tumors have been shown to produce responses by targeting their DNA damage response (DDR) pathways. Although the clinical impact and prevalence of DDR changes in GC remain unknown, evaluating the DDR pathway and its relevance to prognostic and clinical features may have significantly innovative implications for treating patients with GC [35]. In the last decade, immunotherapeutic strategies that inhibit CTLA-4 and PD-1/PD-L1, the well-known immunological checkpoints, have shown remarkable success in the treatment of cancer, especially melanoma and Hodgkin’s lymphoma [10]. Recent studies have shown that compared with monotherapy, combination immunotherapy targeting PD-1 and CTLA-4 is more effective in treating GC [36]. Given that PD-L1, PD-1, and CTLA-4 show a positive correlation with each other in tumor tissues, combining these drugs might show overlapping clinical applications [37]. Therefore, future studies should be aimed at improving the effectiveness of immunotherapy. In this study, we analyzed the prognostic significance and expression status of *C5aR1* in GC. The results depicted that the 10-year OS rate was worse in patients with higher *C5aR1* expression compared with those with low *C5aR1* expression. Functional analysis revealed that the *C5aR1* gene is related to immune processes that might play a key role in inflammatory tumor immune responses, promoting the carcinogenesis and progression of GC. A critical characteristic of inflammatory TME is the upregulation of immune checkpoints that are suppressive in function and are driven by pre-infiltrative tumor-infiltrating immune cells [38]. Therefore, the association of expression of *C5aR1* with immune checkpoint-related genes and immune cells was also analyzed. *C5aR1* showed a negative correlation with the immunological status of TME and a positive correlation with most immune checkpoints in GC, including *PD-L1*, *CTLA-4, LAG-3,* and *PD-1*, which suppress the effector function of T cells and impede anti-tumor immunity. These results reveal that combining anti-C5aR1 and immune checkpoint blockade therapies may increase the effectiveness of immunotherapy in GC. Analysis performed using the CIBERSORT algorithm revealed that the process of recruitment of T cells was significantly downregulated in the high C5aR1 expression group. In addition, *C5aR1* showed a negative correlation with NK cells, CD8+ T cells, and dendritic cells, and the infiltration level of tumor-infiltrating immune cells was significantly reduced. These results indicate that C5aR1 can inhibit the proliferation of CD8+ T cells from promoting immune escape.

Tumor immune infiltration and immune escape are correlated with cancer prognosis and treatment response [39–41]. Previous studies have reported that the relationship between *C5aR1* expression and tumor immune infiltration may enhance tumor immune escape through a dysfunctional T cell phenotype [42]. In this study, TIDE [23] scores were estimated, and their distribution was visualized on a box plot to evaluate the immune escape potential of *C5aR1* in GC. The results revealed that immune escape potential was elevated in the high C5aR1 expression group, which was verified via IHC analysis. Therefore, we hypothesized that anti-C5aR1 treatment could inhibit immune escape and hence enhance immunotherapeutic efficacy in GC.

Although our evidence suggests that C5aR1 shapes a noninflammatory TME and mediates immune evasion in GC, there are some limitations in our study. Our exploration of the role of complement family genes in GC was based on data available in the GEO and TCGA databases, and some of our findings were validated by our validation cohort. However, we did not perform animal experiments to confirm the link between C5aR1 and immune cells infiltrating in TME, which pinpoint the direction for our future work.

## Conclusion

*C5aR1* expression level is elevated in GC tissues compared to normal gastric tissues. Patients with high *C5aR1* expression levels have worse 10-year OS compared to those with low *C5aR1* expression. From the results of functional annotation and pathway enrichment analyses, C5aR1 is primarily involved in inflammatory and immune responses. C5aR1 can inhibit the proliferation of CD8+ T cells and promote immune escape. Using immune phenomenon scores, we found that anti-CTLA-4 immunotherapy was more effective in the low complement score group. This study offers novel insights into the immunotherapy of GC.

## Acknowledgments

We thank Bullet Edits Limited for language editing and proofreading of the manuscript and open databases such as TCGA, GEO, GEPIA, Kaplan–Meier Plotter, and TIDE for providing the platform and datasets.

## Supplemental Data

Raw data can be found at the following link: https://www.jianguoyun.com/p/DaCcEvUQx5WACximstwEIAA.
